# The Etiology of Cleft Palate Formation in BMP7-Deficient Mice

**DOI:** 10.1371/journal.pone.0059463

**Published:** 2013-03-14

**Authors:** Thaleia Kouskoura, Anastasiia Kozlova, Maria Alexiou, Susanne Blumer, Vasiliki Zouvelou, Christos Katsaros, Matthias Chiquet, Thimios A. Mitsiadis, Daniel Graf

**Affiliations:** 1 Orofacial Development and Regeneration, Institute of Oral Biology, Center for Dental Medicine, University of Zurich, Zurich, Switzerland; 2 Department of Orthodontics and Dentofacial Orthopedics, School of Dental Medicine, University of Bern, Bern, Switzerland; 3 Department of Histology and Embryology, Medical School, National and Kapodistrian University of Athens, Athens, Greece; Team 'Evo-Devo of Vertebrate Dentition', France

## Abstract

Palatogenesis is a complex process implying growth, elevation and fusion of the two lateral palatal shelves during embryogenesis. This process is tightly controlled by genetic and mechanistic cues that also coordinate the growth of other orofacial structures. Failure at any of these steps can result in cleft palate, which is a frequent craniofacial malformation in humans. To understand the etiology of cleft palate linked to the BMP signaling pathway, we studied palatogenesis in *Bmp7*-deficient mouse embryos. *Bmp7* expression was found in several orofacial structures including the edges of the palatal shelves prior and during their fusion. *Bmp7* deletion resulted in a general alteration of oral cavity morphology, unpaired palatal shelf elevation, delayed shelf approximation, and subsequent lack of fusion. Cell proliferation and expression of specific genes involved in palatogenesis were not altered in *Bmp7*-deficient embryos. Conditional ablation of *Bmp7* with Keratin14-Cre or Wnt1-Cre revealed that neither epithelial nor neural crest-specific loss of Bmp7 alone could recapitulate the cleft palate phenotype. Palatal shelves from mutant embryos were able to fuse when cultured *in vitro* as isolated shelves in proximity, but not when cultured as whole upper jaw explants. Thus, deformations in the oral cavity of *Bmp7*-deficient embryos such as the shorter and wider mandible were not solely responsible for cleft palate formation. These findings indicate a requirement for Bmp7 for the coordination of both developmental and mechanistic aspects of palatogenesis.

## Introduction

Secondary palate forms from paired vertically oriented outgrowths of the maxillary processes called palatal shelves. In a coordinated manner, the palatal shelves expand and elevate to a horizontal position above the tongue, approximate and finally fuse with each other at the midline. Deregulation in any of these developmental steps may result in cleft-palate, a common birth defect in humans. The palatal structures are composed of cranial neural crest-derived mesenchyme and pharyngeal ectoderm [1], [2]. Interactions between these two tissues are essential for growth and fusion of the palate. A number of transcription factors (e.g. Msx1, Tbx22, Irf6) and signaling molecules such as members of the TGFβ (Transforming Growth Factor beta) superfamily have been implicated in the process of secondary palate formation [3], [4], [5].

Bone Morphogenetic Proteins (BMPs) are evolutionarily conserved secreted signaling molecules that belong to the TGFβ superfamily. BMPs are critical for correct patterning of many embryonic primordia, including the orofacial structures [5], [6], [7]. Based on their homology, BMPs can be divided into two subfamilies: the subfamily of BMP2 and BMP4, which are orthologues of *Drosophila melanogaster* dpp, and the subfamily of BMP5, BMP6, BMP7 and BMP8, which are orthologues of gbb/60A [8]. BMPs signal through a receptor complex that consists of two type I serine-threonine kinase receptors, i.e. Activin receptor-like kinase (Alk)1, Alk2 (also known as AcvrI, ActRI, or ActRIA), Alk3 (BmprIa) or Alk6 (BmprIb) and two type II receptors (BmprII or ActRII) [9]. BMP signaling activity *in vivo* is highly regulated at several levels of the pathway, including extracellularly where secreted BMP-binding proteins like Noggin, Chordin, and Gremlin act as BMP antagonists [10]. BMP ligands have diverse binding affinities for receptors and antagonists [11], which contributes to the precise spatio-temporal regulation of BMP biological activity *in vivo.* However, the cell-type specific requirements for individual members of the BMP family in tissue interactions are still poorly understood.

Several BMPs are expressed in facial primordia [12], [13] and in the developing palatal shelves [14], [15]. Bmp2 and Bmp4 regulate proliferation in the mesenchyme through a gene network involving *Msx1* and *Shh* [16]. Inactivation in the facial primordia of the type 1 BMP receptor Alk3, to which Bmp2 and Bmp4 bind with high affinity, results in cleft lip and palate, and tooth agenesis [17]. The defect is associated with decreased cell proliferation in the mesenchyme of the maxillary process and abnormal anterior-posterior patterning of the palate. Similarly, inactivation of Alk3 in cranial neural crest cells leads to cleft formation due to defective mesenchymal cell proliferation [18]. In contrast, neural crest specific ablation of Alk2 results in cleft palate due to palatal shelf elevation failure [19]. These findings illustrate the importance for BMP signaling in palate formation at various developmental stages.

There is evidence from humans, that BMP7 is also a key factor for development of the secondary palate. In a recent report a mutation in *BMP7* was identified in an 8 year-old boy who presented with absence of eyes, hearing loss, high palate with a mild cleft and crowded teeth [20]. Similarly, *Bmp7*-deficient mouse embryos show numerous orofacial abnormalities, including absence of eyes, discontinuity of the cranial and acoustic bones, micrognathia, missing or malformed/misplaced teeth, and cleft palate with 100% penetrance [21]. *Bmp7* is expressed in several orofacial structures, most prominently in the epithelium and underlying mesenchyme at the tip of the palatal shelves.

In this study we investigated which orofacial structures require Bmp7 for secondary palate formation. We find that the combined absence of Bmp7 in several orofacial structures is necessary to cause the cleft palate phenotype.

## Methods

### Animals

The *Bmp7^wt/Δ^* allele used in this study was derived by deleting a conditional *Bmp7^wt/flx^* allele by Cre-mediated recombination in the germline [22]. Bmp7 heterozygous null mice (*Bmp7^wt/^* ^Δ^) were intercrossed to obtain *Bmp7* ^Δ*/*Δ^ embryos and control littermates. Genotyping of embryos was carried out by allele-specific PCR as described [22]. The *Bmp7lacZ* allele [23] has been described elsewhere. For conditional deletion, the K14-Cre [24] or wnt1-Cre [25] lines were used. All mouse lines were backcrossed for more than 8 generations into the C57Bl6/J background. Mice were maintained at the animal facilities of the University of Zurich. Animal experiments were approved by the local veterinary authorities (permit 98/2011, Veterinäramt Zürich) in compliance with Swiss federal law (TSchG, TSchV) and cantonal by-laws in full compliance with the European Guideline 86/609/EC. This authority approval also included ethical approval. Embryos were obtained by timed mating, and E0.5 was considered as the morning where the vaginal plug was seen.

### Histological staining of sections

Cryosections of paraformaldehyde (PFA)-fixed embryonic heads were stained with Alcian Blue solution (1% w/v in 3%v/v aqueous acetic acid, pH 2.5), rinsed and counterstained with Fast Red (0.1% w/v). Sections were rinsed, refixed in 4% PFA, and mounted with water-based Mowiol 4–88 (Sigma).

### LacZ staining of embryonic tissues or sections

Tissues from heterozygous *Bmp7lacZ* (*Bmp7^wt/lacZ^*) embryos (expressing β-galactosidase under the control of the *Bmp7* promoter) were stained with X-gal to identify the location of *Bmp7* expression. Briefly, tissue fragments or OCT (BDH)-embedded palate sections were fixed in 2% formaldehyde, 0.2% glutaraldehyde, 0.01% sodium deoxycholate, 0.02% Nonidet-P40 (NP40) in PBS for 5 min, washed with 2 mM MgCl_2_, and stained at 37°C in the dark overnight in X-gal staining solution, which contained 0.1 M phosphate pH 7.3, 2 mM MgCl_2_, 0.01% sodium deoxycholate, 0.02% NP40, 5 mM K_3_[Fe(CN)_6_], 5 mM K_4_[Fe(CN)_6_] supplemented with 1 mg X-Gal (Promega)/ml. On the following day, sections were washed and refixed in 4% PFA. Sections were mounted with water-based Mowiol 4–88 (Sigma) and documented using a Leica DM-E microscope equipped with a Leica DFC290 camera. Whole mount staining was documented on a Leica M9.5 stereoscope equipped with a Leica DFC290 camera. Experimental results were obtained from at least three independent samples.

### *In situ* hybridization

All embryos were collected at appropriate time points, fixed in 4% PFA in PBS overnight at 4°C, and subsequently washed in PBS. Embryo heads equilibrated in 30% sucrose were embedded and frozen in cryomedium (Tissue-Tek® O.C.T.™ Compound, Weckert Labortechnik). In situ hybridization was performed on 12 μm thick cryosections with digoxigenin-labeled antisense RNA probes prepared using the MAXIscript® Kit (Ambion) or the Digoxygenin Labeling and Detection Kits (Roche) according to protocols provided by the manufacturers and standard protocols. Experimental results were obtained from at least three control and mutant embryos, respectively.

### Immunohistochemistry

Immunohistochemical experiments were performed on frontal cryosections from embryonic heads obtained as above. Cell proliferation was assessed using a primary rabbit anti- phospho-histone H3 antibody (Santa Cruz Laboratories) and a secondary biotinylated donkey anti-rabbit IgG antibody (Southern Biotech). Signal detection was carried out using the PK-6100 ABC Peroxidase kit (Vector Laboratories) and the DAB Peroxidase Substrate Reagent (Vector Laboratories) as an enzyme substrate. Apoptotic cell death was assessed by staining cryosections using the TUNEL Apoptosis Detection System (GenScript) following the manufacturer's instructions. For Keratin14 staining an anti-K14 antibody (Enzolife Science) was used as the primary antibody. The endogenous peroxidase activity was in each case quenched by incubating the sections in 1 mM β-D (+) glucose, 0.01 mg glucose oxidase, 0.005% sodium azide in PBS at 37°C for 1 h. The number of cells labeled with either anti-phosphohistone H3 antibody or TUNEL kit was recorded for equivalent areas of coronal palatal shelf sections (defined by a line between the lateral nasal wall and the hinge region) from the anterior, middle and posterior regions of the secondary palate. Data were collected from three pairs of mutant and control littermates at each developmental stage. Student's t-test was used to analyze the significance of differences and a P-value of less than 0.05 was considered statistically significant.

### Whole mount cartilage and skeletal staining

E14.5 whole embryos were fixed in 95% ethanol overnight. Staining was carried out with Alcian Blue (0.4% Alcian Blue 8GS/74% ethanol/20% glacial acetic acid) and Alizarin Red (0.5 mg/ml). Tissue was treated successively with 1% KOH, 2% KOH and rinse in 0.25% KOH. Clearing was carried out in consecutive changes of glycerol (20%, 33%, 50%), and the embryos were stored in 50% glycerol/0.25% KOH.

The heads of whole mount stained of *Bmp7^Δ/Δ^* or control littermate embryos were isolated, positioned and photographed on a Leica M9.5 stereoscope equipped with a Leica DFC290 camera at identical magnification. Experimental results were obtained from at least three control and mutant embryos, respectively.

### *In vitro* palate fusion assays

Palatal shelves from E14.5-E16.5 *Bmp7^Δ/Δ^* or control littermate embryos were dissected. The palatal shelves from control embryos at this stage had elevated but were still not apposed closely enough for fusion having occurred. The microdissected palatal shelves were cultured as Trowell type organ cultures on 0.65-mm stainless steel grids separated from the grid by a filter membrane (Nucleopore 25 MM/0.05 μM, Whatman). The paired shelves were placed with their future fusing edges in close apposition and without distortion of the tissue shape, while their oral side was facing up. They were cultured at 37°C with 5% CO_2_ using Dulbecco's modified Eagle's Medium (DMEM) supplemented with 1% streptomycin (changed every 24 h). Pictures were taken at several time points of culture. At the end of the incubation period, the cultures were fixed in 4% PFA, dehydrated, and embedded in paraffin. Selected sections were deparaffinised and stained for K14 as described above. Experimental results were obtained from at least three control and mutant embryos, respectively.

### Organotypic palate explant culture

For the organotypic explants culture we adapted a previously described method for culturing the entire maxillary region with attached palatal shelves [26] under serum free conditions. For this the mandibular region including the tongue was removed from the head of E12.5 or E13.5 embryos and the brain was dissected at the level of the eyes to leave the entire maxillary region with palatal shelves attached. The explants were cultured in suspension in DMEM supplemented with 10% Serum Replacement (Invitrogen), glutamine, antibiotics (penicillin and streptomycin) and 4 mg/ml BSA. Cultures were maintained under gentle movement in an incubator at 37°C in a gas mixture of 5%CO_2_/50%O_2_/N_2,_ for 66–72 h whereby the medium was replaced after 48 hrs. After culture the explants were briefly fixed in 0.2% glutaraldehyde/2% formaldehyde and processed for whole mount lacZ staining. (N _control_ >10, N_Bmp7_^Δ/Δ^ = 5).

## Results

### *Bmp7^Δ/Δ^* embryos exhibit delayed palatal shelf elevation

**Figure 1:**
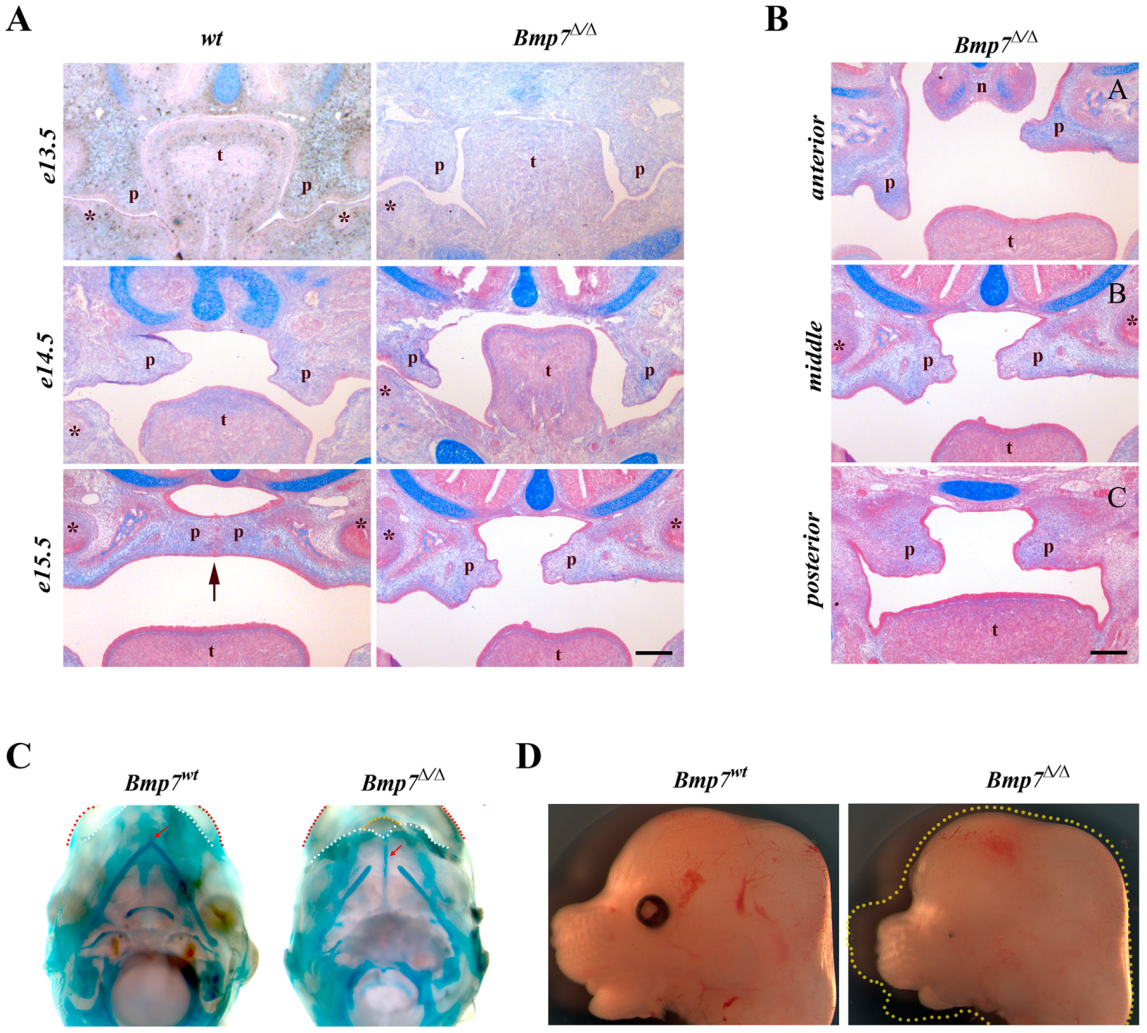
Histological analysis of secondary palate development and morphological features of *Bmp7-*deficient mouse embryos. (A) Coronal sections from the middle region of the palate at E13.5-E15.5. Whereas the palatal shelves in both *Bmp7^wt/wt^* and *Bmp7^Δ/Δ^* embryos were similar in shape at E13.5, the shelves of the *Bmp7^Δ/Δ^* embryos at E14.5 remained vertically oriented when compared to *Bmp7^wt/wt^* littermate embryos. Note the increased tongue height in *Bmp7^Δ/Δ^* embryos. At E15.5 the palatal shelves of *Bmp7^Δ/Δ^* embryos were in the process of reorientation, while the shelves of *Bmp7^wt/wt^* littermates already had started to fuse along their medial edges (arrow). (B) Coronal sections along the A-P axis of the developing palates at E15.5 stained with Alcian Blue and Fast Red demonstrate regional differences in palatal shelf reorientation in the *Bmp7^Δ/Δ^* embryos. The delay in reorientation is most evident in the anterior region. The mid-palatal region was defined by the presence of the 1^st^ molar tooth bud (*) on the sections. n; nose, p; palatal shelf, t; tongue, *Scale bar: 100* *μm.* (C) Ventral views of Meckeĺs cartilage (stained with Alcian Blue) at E14.5 reveal that its halves are significantly shorter and fail to fuse in the anterior region in *Bmp7^Δ/Δ^* embryos, resulting in a shorter mandible (white dotted lines) with a protruding tongue (yellow dotted line). Upper lips are marked (red dotted lines). (D) Side views of *Bmp7^Δ/Δ^* and *Bmp7^wt/wt^* littermate heads show that the growth of the nasal prominence is also stunted in the mutant in addition to the shorter mandible. Yellow dotted outline in image of mutant embryo corresponds to wt littermate.

To assess how the lack of Bmp7 affects the development of the palatal shelves, we compared stained coronal sections of both *Bmp7^wt/wt^* and *Bmp7^Δ/Δ^* embryos during key stages of palatogenesis. In comparison to the wild-type littermates, *Bmp7^Δ/Δ^* embryos displayed an apparent delay in shelf elevation. Specifically, at E13.5 the mutant palatal shelves seemed morphologically normal and correctly positioned in relation to the tongue when compared to their wild-type littermates (Fig. 1A). At stage E14.5, while the wild-type palatal shelves had nearly completed their reorientation and lay above the dorsal surface of the tongue, the *Bmp7^Δ/Δ^* littermate palatal shelves were still in the vertical position (Fig. 1A). By E15.5 the wild-type palatal shelves were horizontally positioned in relation to the tongue and had made contact with each other along their medial edges initiating palatal fusion. At the same developmental stage (E15.5) the *Bmp7^Δ/Δ^* palatal shelves were in the process of reorientation, and thus showed an apparent delay in elevation of at least 24 hrs (Fig 1A) when compared to their wild-type littermates. Elevation of the *Bmp7^Δ/Δ^* palatal shelves was asymmetric and occurred over a longer time period. Analysis of the developing palate in *Bmp7^Δ/Δ^* embryos at E15.5 along the A-P axis revealed regional differences in the timing of palatal shelf reorientation. The delay was most prominent in the anterior region. In the representative example shown in Fig. 1B, one palatal shelf was still vertical and the other one had acquired a horizontal orientation. In the middle region, the delayed palatal shelf had started its reorientation, whereas in the posterior region both palatal shelves had assumed a similar horizontal position above the tongue but remained apart (Fig. 1B). When the fusion of the palatal shelves was completed in the wild-type embryos at E16.5, the shelves in the *Bmp7^Δ/Δ^* littermates had attained their horizontal positions above the dorsum of the tongue all along the A-P axis but remained separated from each other with no sign of contact at the midline, resulting in cleft of the secondary palate at birth.

Bmp signaling plays an important role in skeletogenesis of the head [19], [27], and *Bmp7^Δ/Δ^* embryos show numerous craniofacial abnormalities, including discontinuity of the cranial and acoustic bones and micrognathia [21]. Growth retardation of Meckel's cartilage and mandible can interfere with forward displacement of the tongue and in consequence disturb palatal shelf elevation [28]. Skeletal preparations at E14.5 revealed that the length of Meckeĺs cartilage in the *Bmp7^Δ/Δ^* embryos was significantly reduced and failed to fuse at the anterior end when compared to control littermates (Fig. 1C). However, growth retardation was not restricted to the mandible, since development of the maxilla and the nasal prominence were equally affected (Fig. 1D). This indicates that lack of Bmp7 affects the development and growth of the orofacial region in general.

**Figure 2:**
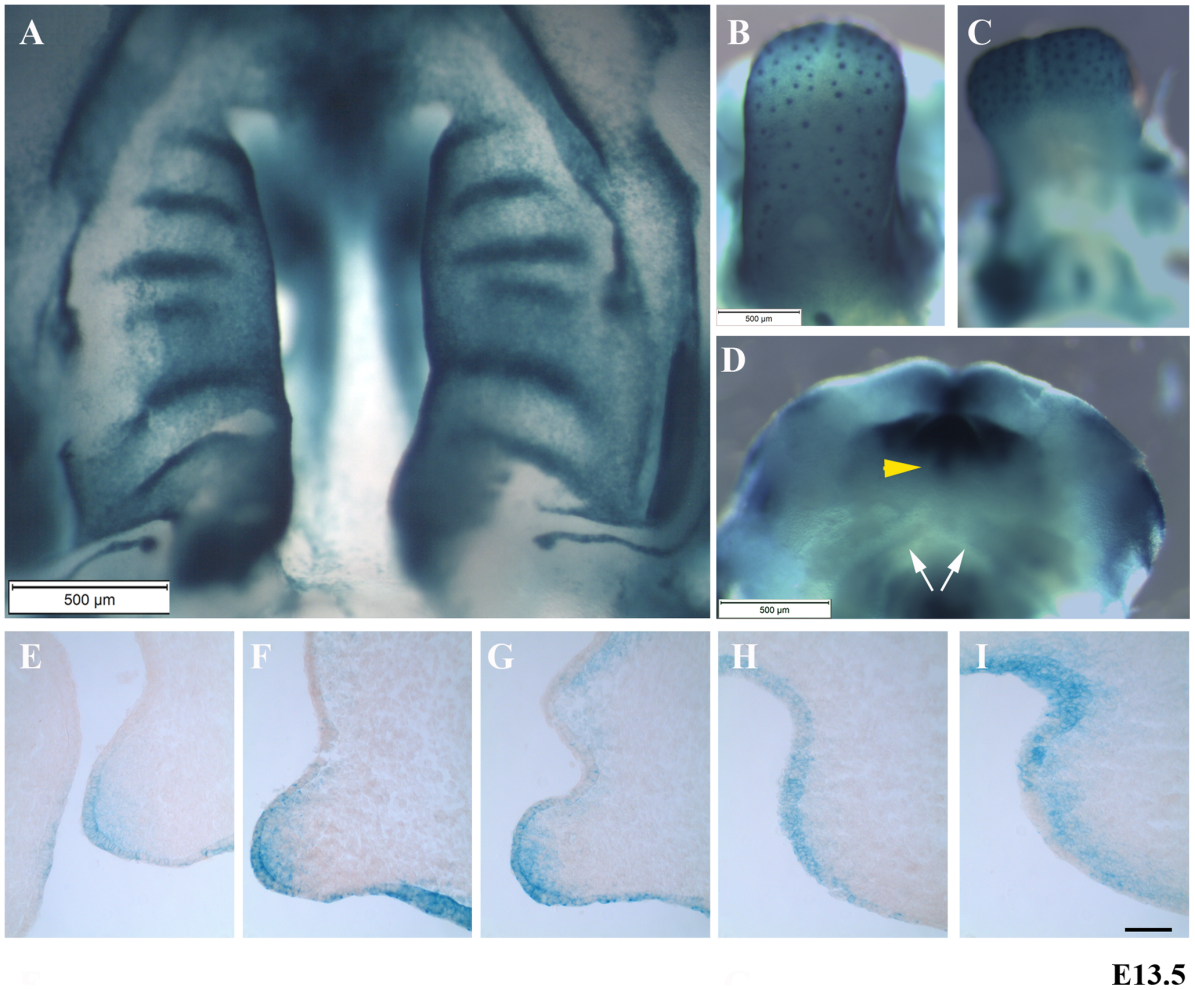
Bmp7 expression in *Bmp7-lacZ* mouse embryos at stage E13.5. Widespread transcriptional activity of *Bmp7* is revealed by lacZ staining in the developing palate (A), the tongue (B, C), and the lower lip (D). Note the strong lacZ staining in the developing palatal rugae, the gustatory follicles and the midline of the lower lip (arrowhead). Weak *Bmp7* expression is observed in Meckel's cartilage (arrows). (E–I) *Bmp7* transcriptional activity appeared to be dynamic with some regional anteroposterior differences and could be detected both in palatal epithelium and mesenchyme (Fig. 2E–I; E anterior, I posterior). *Scale bar, 50* *μm.*

To assess the expression of *Bmp7* in orofacial structures and in particular the palatal shelves at the onset of palatal shelf elevation, E13.5 *Bmp7-lacZ* embryos were stained with X-gal. An intraoral view of the palatal shelves shows widespread expression of *Bmp7*, with a clear demarcation of the developing rugae and the medial edges (Fig. 2A). Similarly, *Bmp7* expression in the tongue was widespread and strongest in the developing taste buds (Fig. 2B), but apparently absent from the tongue muscle (Fig 2C). The tip of the developing lower lip showed strong expression of *Bmp7,* whereas weak staining was observed in structures surrounding the developing Meckel's cartilage (Fig. 2D). Serial coronal cryosections of E13.5 embryonic heads showed dynamic expression of *Bmp7* in an anterior-posterior (A–P) direction (Fig. 2, E–I). *Bmp7* was expressed in the epithelium along the entire length of the palatal shelves, while the adjacent mesenchyme in the anterior two-thirds also showed *Bmp7* expression. The mesenchymal *Bmp7* expression was largely absent from the posterior third of the secondary palate (Fig. 2H), but reappeared at the posterior most edges of the shelf tissue (Fig. 2I).

### Normal expression of epithelial marker genes and genes related to palatal shelf adhesion and fusion in *Bmp7^Δ/Δ^* palatal shelves

**Figure 3:**
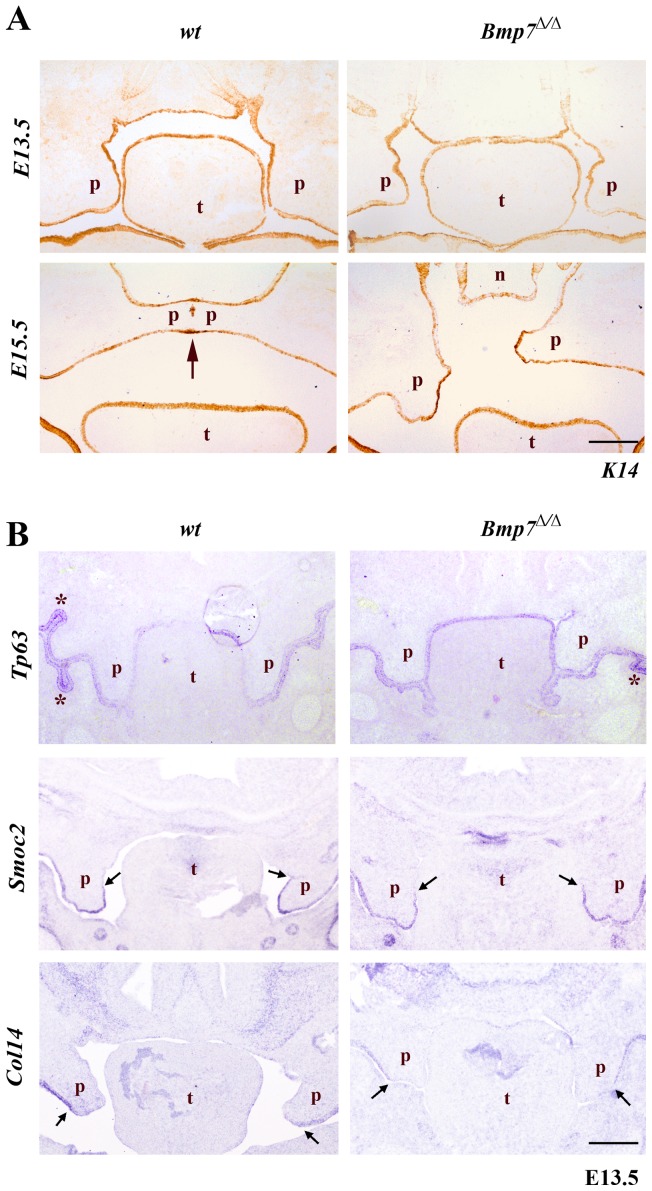
Expression of epithelial marker genes. (A) Cryosections were stained with anti-K14 antibody marking the oral epithelium. At E13.5 the palatal shelves are very similar for both the *Bmp7^wt/wt^* and *Bmp7^Δ/Δ^* mouse embryos. At E15.5 the *Bmp7^wt/wt^* shelves fuse along their contact areas at the midline; there are still remnants of epithelium (stained brown due to presence of K14) along the medial epithelial seam. At the same developmental stage the palatal shelves of the *Bmp7^Δ/Δ^* littermates have not achieved contact along their medial edges, and the presumptive medial edge epithelia persists. (B) The expression of *Tp63* specific for oral epithelium and of genes *Smoc2* and *Collagen14* that are indicative of regional specification of the palatal epithelium was also unaffected in *Bmp7^Δ/Δ^*, as shown by in situ hybridization. *Scale bar: 100* *μm.*

As the expression of *Bmp7* in the palatal shelf shelves was strongest in epithelial structures and the underlying mesenchyme, and since Bmp signaling regulates epithelial-mesenchymal interactions in various systems, we assessed next whether loss of *Bmp7* would affect the development and differentiation of the palate epithelium. At E13.5 keratin 14 (K14) marks the entire oral epithelium including the palatal shelves in both wild type and *Bmp7^Δ/Δ^* embryos (Fig. 3A). At E15.5, the midline fusion of the palatal shelves in wild-type embryos manifests itself by the gradual disappearance of the midline epithelial seam (MES) between the contacting shelves. After initial contact, K14 expression still marks the intact epithelial layer of the seam, and as the epithelial seam starts to disappear gradually, K14 antibody labels its remnants (Fig. 3A) [29]. In *Bmp7^Δ/Δ^* embryos, the palatal shelves do not make contact and K14 remains expressed in the uninterrupted epithelial layer of the unfused palatal shelves (Fig. 3A).

The extracellular matrix proteins Smoc2 and Collagen14 are differentially expressed in various regions of the palatal epithelium. Smoc2 is a small Ca-binding protein that has recently been implicated in oligodontia in humans [30]. Collagen14 is a FACIT (fibril associated collagen with interrupted triple helix) involved in fibrillar collagen assembly [31]. In E13.5 wild type embryos *Smoc2* mRNA is found in the lateral and medial-distal epithelium of the palate, whereas *collagen14* is expressed only in the lateral palate epithelium (Fig. 3B). *Bmp7^Δ/Δ^* embryos showed an identical expression pattern of these genes, indicating that maintenance of regional epithelial identity in *Bmp7^Δ/Δ^* embryos is not affected (Fig. 3B).

**Figure 4:**
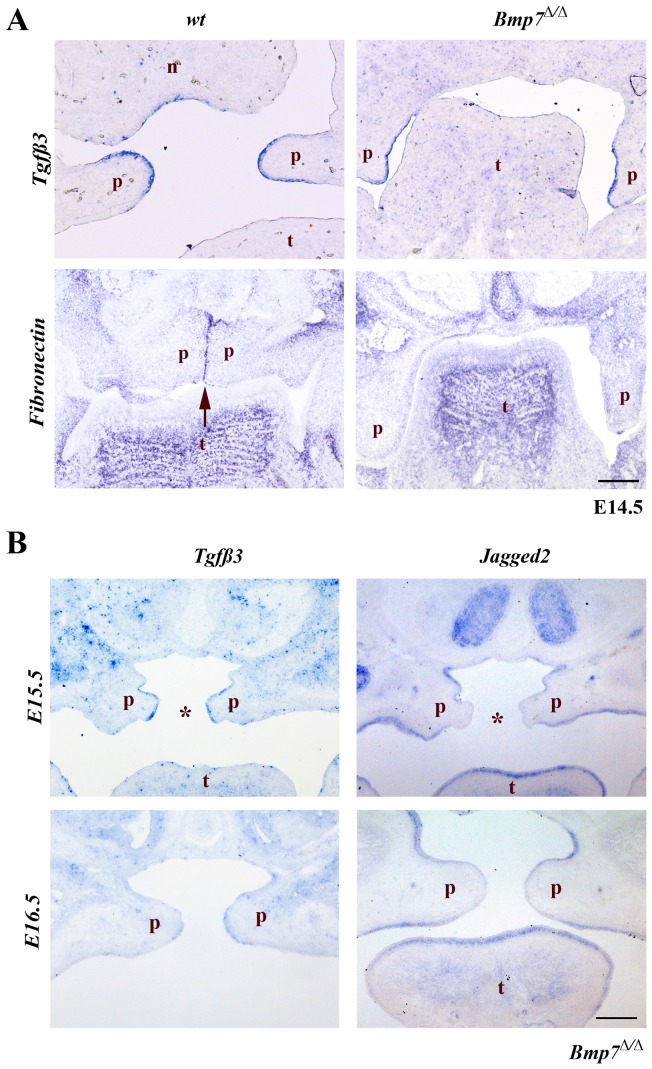
Expression of genes involved in palatal shelf adhesion and fusion. In situ hybridization for *Tgfβ3*, *Jagged2* and *fibronectin (Fn)*. (A) The expression of *Tgfβ3* at E14.5 is similar in both *Bmp7^wt/wt^* and *Bmp7^Δ/Δ^* embryos and limited to the presumptive medial edge epithelium. Expression of *Fn* is evident in the medial edge epithelium of the palatal shelves in *Bmp7^wt/wt^* embryos, but absent from corresponding regions of the *Bmp7^Δ/Δ^* palatal shelves at the same developmental stage. (B) In the *Bmp7^Δ/Δ^* embryos expression of *Tgfβ3* at the medial edge epithelium of the palatal shelves is still strong at E15.5 and starts disappearing at E16.5. In the *Bmp7^Δ/Δ^* embryos a specific down-regulation of *Jagged2* expression at the presumptive MEE is noted at stages E15.5 and E16.5. *Scale bar: 100* *μm.*

To determine whether the cleft palate in *Bmp7^Δ/Δ^* embryos was primarily due to the inability of the palatal shelves to reach a critical proximity to each other or whether it is caused by an inherent inability to fuse, we next tested for the expression of *Tgfβ3*, which is normally expressed specifically in the medial edge epithelial cells before palatal shelf fusion and is critical for the fusion process [32]. Once the medial epithelial seam has formed *Tgfβ3* expression quickly ceases [33]. *Tgfβ3* was expressed in the presumptive MEE [34] of *Bmp7^Δ/Δ^* mice at E14.5 comparably to wild type embryos (Fig. 4A).

Fibronectin (Fn) is an extracellular matrix protein expressed on the outer surface as well as in the intercellular space of MEE cells, and is up-regulated upon contact of the palatal shelves in response to Tgfβ3 [35]. Whereas at E14.5 *Fn* is strongly expressed in the MES of wild type palatal shelves, it was not induced in the respective region of the *Bmp7^Δ/Δ^* palatal shelves. This demonstrates that cellular events that precede palatal fusion but require shelf contact are not occurring in *Bmp7* deficient embryos (Fig. 4A).

Interestingly, expression of *Tgfβ3* in the presumptive MEE of *Bmp7^Δ/Δ^* embryos was still evident at E15.5 and began to decrease at E16.5 (Fig. 4B), suggesting that the palatal shelves might be able to fuse until at least E15.5. In control littermates, *Tgfβ3* was gradually down-regulated along with the disappearing midline epithelial seam in the course of palatal fusion (not shown).

Jagged2 is expressed along all palate epithelium to prevent the fusion of the oral epithelial structures and ceases its expression in the MEE just prior to fusion of the palatal shelves [36], [37] Similar to *Tgfb3, Jagged 2* was normally down-regulated in the presumptive MEE cells of the *Bmp7^Δ/Δ^* embryos and remained absent in this epithelial region at E15.5 and E16.5 even though no palatal shelf contact was made (Fig. 4B). These results indicate that in *Bmp7^Δ/Δ^* palatal shelves, MEE cells are specified at the correct time point despite the delay in shelf elevation.

### *Bmp7^Δ/Δ^* palatal shelves fuse *in vitro*

**Figure 5:**
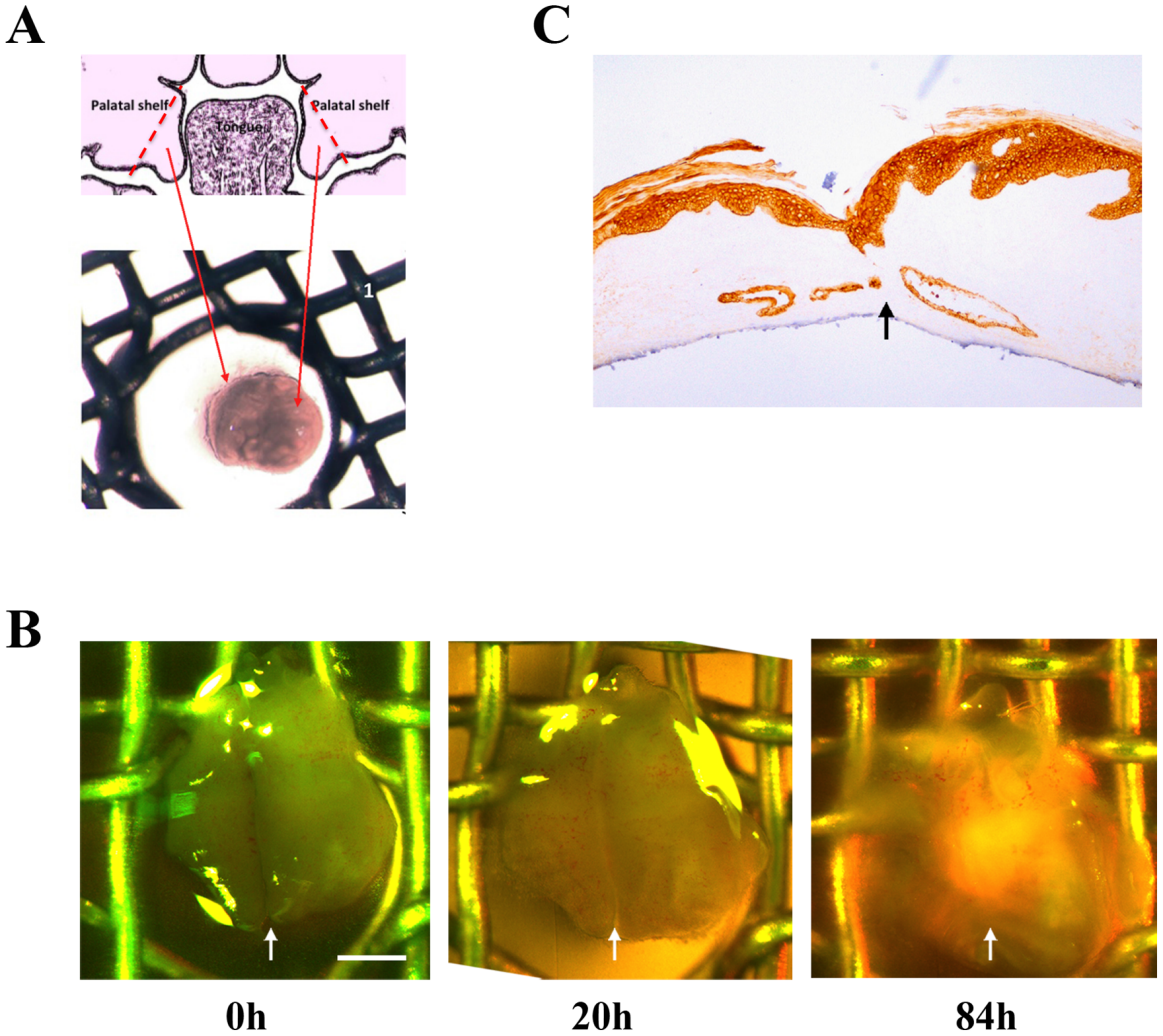
*In vitro* cultures of palatal explants from *Bmp7* show more *^Δ^* ^***/****Δ*^ embryos. (A) Pairs of palatal shelves dissected from developmental stages E14.5 to E16.5 were placed in apposition *in vitro and* cultured. The results showed that *Bmp7^Δ/Δ^* palatal shelves retain the ability to fuse even at E16.5. (B) An example of a *Bmp7^Δ/Δ^* palatal shelf from E14.5 is shown at the start (0 h), after 20 h and 84 h of culture. Fusion at the midline is visually evident at 84 h and the formation of palatal rugae indicates continuing palatal development. *Scale bar: 500* *μm* (C) Explants were sectioned coronally and stained for Keratin14. Continuity of the epithelial layer and disappearance of the medial epithelial seam indicate successful fusion.

In order to confirm that in the *Bmp7^Δ/Δ^* mice the palatal shelves were able to fuse, we carried out *in vitro* palatal fusion assays using pairs of dissected shelves from *Bmp7^Δ/Δ^* embryos at E13.5-E16.5 (Fig. 5A). As a control we included paired palatal shelf explants from E13.5 or E14.5 wild type embryos. The tissue cultures were visually examined at various time points to confirm viability and shelf fusion.

The results of these assays showed that the palatal shelves of *Bmp7^Δ/Δ^* embryos have retained the ability to fuse in culture when placed in close proximity (Fig. 5B). Fusion of palatal shelves was obtained at all the developmental stages tested (up to E16.5). Fusion and the complete disappearance of the midline epithelium were confirmed by sectioning and staining the fused palates with antibody to K14 (Fig. 5C). These findings suggest that the epithelial component of the mutant palatal shelves undergoes normal differentiation and that the palatal shelves are fusion competent at least up to 48 hrs after the time point when fusion normally occurs.

### Normal expression of mesenchymal marker genes and normal proliferation in the palatal shelves of *Bmp7^Δ/Δ^* embryos

**Figure 6:**
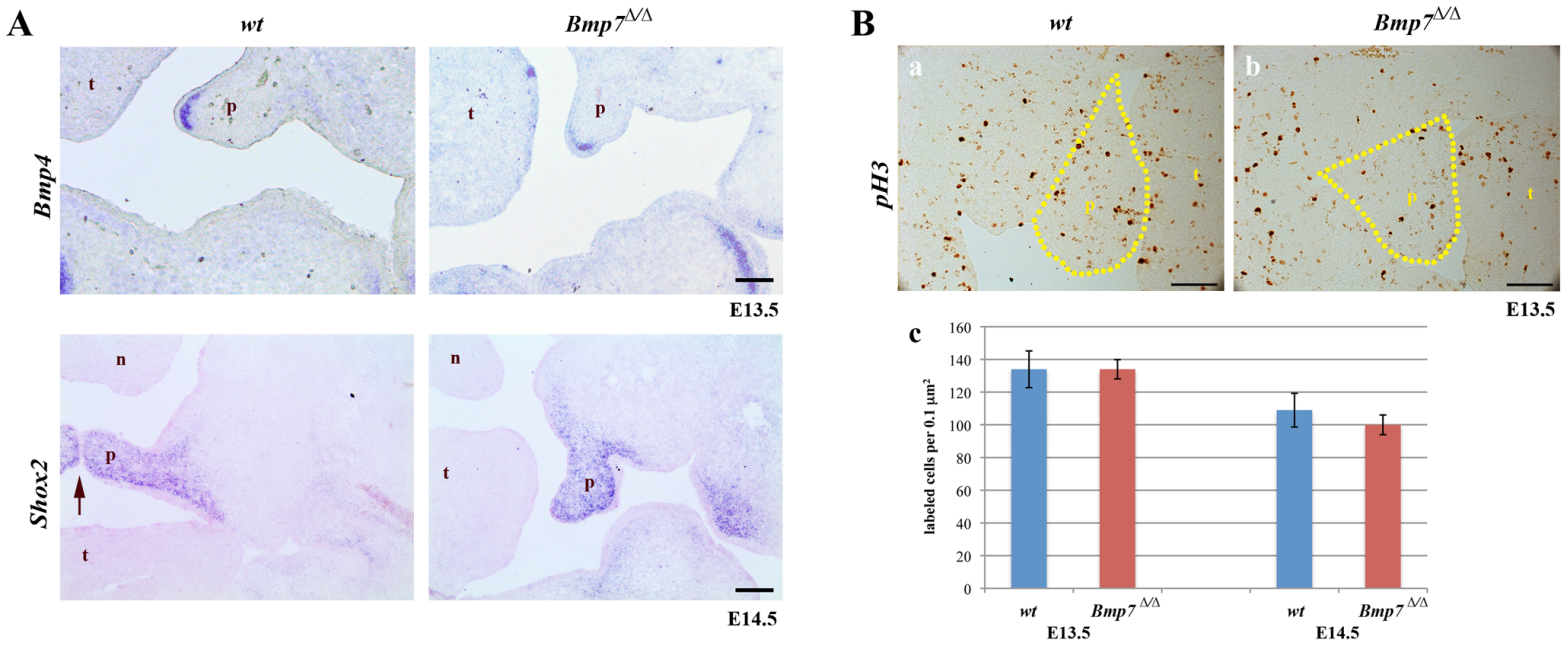
Expression of mesenchymal marker genes and proliferation in palatal shelves at developmental stages E13.5-E14.5. (A) In situ hybridizations for *Bmp4* at E13.5 and *Shox2* at E14.5 reveal similar expression in *Bmp7^wt/wt^* and *Bmp7^Δ/Δ^* embryos. At E14.5 *Bmp7^wt/wt^* palatal shelves were elevated and in approximation (arrow). (B) To identify proliferating cells, *Bmp7^wt/wt^* and *Bmp7^Δ/Δ^* palatal shelves were stained for phosphohistone H3. Coronal sections of E13.5 *Bmp7^wt/wt^* (a) and *Bmp7^Δ/Δ^* (b) palatal shelves (stippled areas) are shown (positive cells are dark brown). Comparison between wild type and knock-out embryos did not reveal any significant difference in the number of proliferating cells at stages E13.5 and E14.5 (c). n; nose, p; palatal shelf, t; tongue, *Scale bar: 100* *μm.*

Next, we investigated gene expression domains of components required for normal growth and patterning of the palatal mesenchyme prior to shelf elevation. We focused on genes normally expressed in the anterior region, such as *Bmp4* and *Shox2*, a SHOX family member of paired-related homeobox gene [38], as this region appeared more strongly affected by the lack of Bmp7. Both *Bmp4* and *Shox2* have established roles in growth and differentiation of the anterior palate [16], [38], [39]. *Bmp4* showed a localized mesenchymal expression at the edges of palatal shelves at E13.5 and no difference between control and mutant was evident (Fig. 6A). Similarly, *Shox2* appeared normally expressed in the anterior region at E14.5. (Fig. 6A). The expression of other anterior markers such as the mesenchymal *Msx1*, were equally unaltered in the mutant palatal shelves (not shown). This indicated that regional specification and cytodifferentiation of the palatal mesenchyme were not dependent on Bmp7.

Bmp signaling has mitogenic effects in palate mesenchyme [17], [18], and aberrations in cell proliferation or apoptosis can affect the elevation of the secondary palate in mice [40]. We therefore tested whether loss of Bmp7 affected cell proliferation or cell death at different stages of palatal development (E13.5–E14.5). Cell proliferation within the palatal shelves was determined by immunohistochemistry to identify phosphohistone H3 positive cells. No significant differences in the number of mitotic cells were found at the early stages of palatal development (E13.5 and E14.5) when equivalent areas of control and mutant palatal shelves were compared (Fig. 6B). The same result was obtained for the earlier developmental stage E12.5 (not shown).

Similarly, no differences in the extent of cell death, as examined using the TUNEL assay, were noted. The number of TUNEL-positive cells in control and mutant palatal shelves was small in both the epithelial and the mesenchymal components of *Bmp7^wt/wt^*, as well as *Bmp7^Δ/Δ^* shelves (not shown).

These results establish that loss of Bmp7 does not disturb normal patterning, cell proliferation or apoptosis of the palate mesenchyme.

### Bmp7 in both mesenchymal and epithelial cells contributes to normal palate development

**Figure 7:**
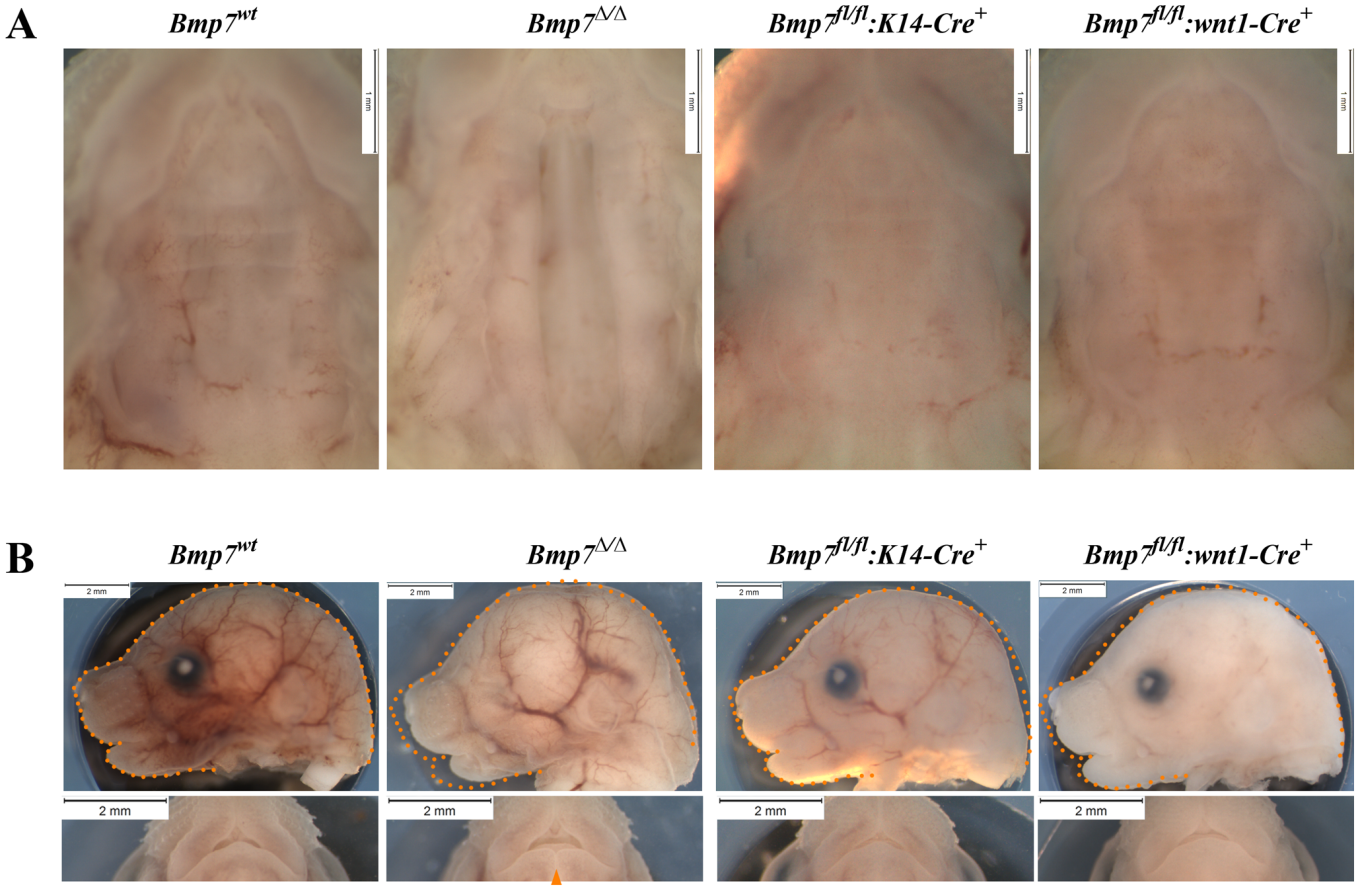
Palate and craniofacial morphology of E16.5 embryos conditionally lacking *Bmp7* in the epithelium or mesenchyme. (A) Ventral views of palates from *Bmp7^wt/wt^*, *Bmp7^Δ/Δ^, Bmp7^fl/fl^*:K14-Cre^+^ and *Bmp7^fl/fl^*:wnt1-Cre^+^ embryos reveal that neither epithelial nor mesenchymal loss of *Bmp7* can recapitulate the cleft palate phenotype of the complete knock-out. (B) Side and ventral aspects of the head show that neither the eye nor the cleft lower lip phenotype (arrowhead) is recapitulated in either conditional mutant. Note, that whereas epithelium-specific deletion of *Bmp7* appeared not to affect snout size (orange dotted outline in image of mutant embryos corresponds to wt littermate) and lower lip, loss of *Bmp7* in the mesenchyme led to a slight reduction in snout size and a dent at the tip of the lower lip.

We next addressed whether *Bmp7* expression was critical in ectodermal or cranial neural crest (CNC) derived ectomesenchymal tissues for normal palate formation. For this, we conditionally deleted *Bmp7* in a tissue specific manner and compared the observed phenotype to *Bmp7^Δ/Δ^* embryos. In Bmp7^fl/fl^:K14-Cre mutant mice, *Bmp7* inactivation was ectoderm-specific, while in Bmp7^fl/fl^:Wnt1-Cre, *Bmp7* deletion was targeted to the cranial neural crest-cells. Complete loss of Bmp7 causes a fully penetrant cleft palate phenotype, as shown for E16.5 embryos in Fig. 7. In addition, disrupted eye development, micrognathia, as well as a cleft lower lip are also clearly visible. Neither epithelial-specific nor neural crest-specific inactivation alone reproduced the cleft palate phenotype observed in the null mutant. Neural crest-specific ablation of *Bmp7* appeared to mildly affect growth of the facial prominence and the development of the lower lip, though not as much as observed in the null mutant.

### Lack of Bmp7 affects palatal shelf elevation directly

**Figure 8:**
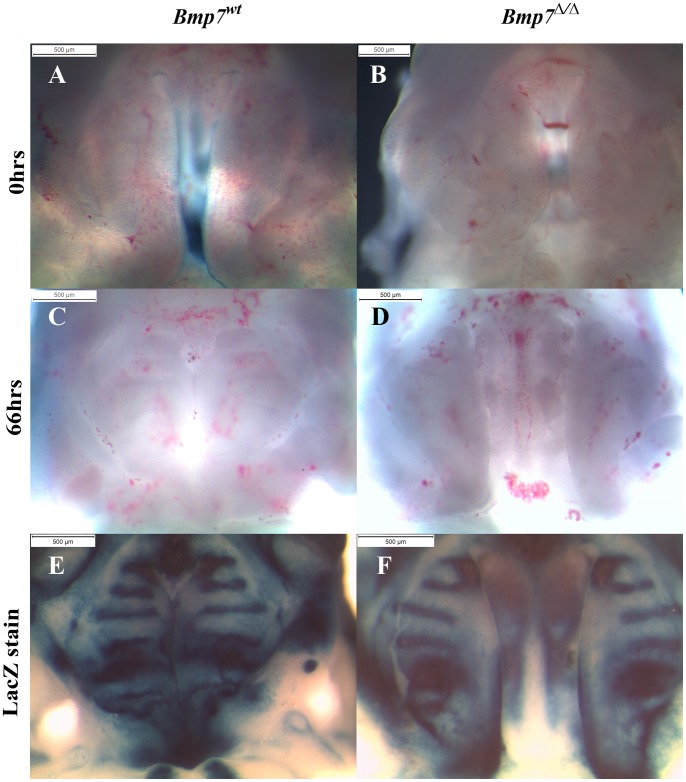
Palate-intrinsic differences in *Bmp7* ^Δ*/*Δ^ mutants revealed by organotypic palate cultures. Ventral view of upper jaw explants from E13.5 *Bmp7^wt/lacZ^* control (A, C, E) or *Bmp7^Δ/lacZ^* (B, D, F) embryos isolated by removing brain, tongue, and mandible prior to culture (A, B), and following culture for 3 days (C, D). Control palatal shelves always showed evidence of reorientation, and fused in approx. half of the cases (C, E). In contrast, mutant shelf elevation was poorer and fusion was never attained (D, F). LacZ staining for *Bmp7* transcriptional activity reveals differences in domains of the developing rugae (E, F).

To address whether the shortened mandible was responsible for the cleft phenotype of *Bmp7^Δ/Δ^* embryos, we established organotypic cultures, where shelf elevation and fusion can proceed in the absence of a possible interference from mandible and tongue. To this aim, the entire maxillary region with attached palatal shelves was isolated at either E12.5 or E13.5 and cultured under high oxygen conditions for 66–72 hrs. Whereas shelf elevation and fusion was observed in more than 50% of the control cultures (wt or heterozygous null), none of the Bmp7 mutant palatal shelves managed to approximate or fuse (Fig. 8). In most cases, the gap between the shelves appeared to increase with time due to the general growth of the orofacial structures. To better illustrate any differences in the shelf appearance, lacZ staining was performed after the culture, showing that the *Bmp7* expression domains in the developing rugae are restricted to more lateral areas in the mutant palates.

These studies indicate that the palatal shelves *per se* are affected in the absence of Bmp7, and that the protruding tongue and retruded mandible are not solely responsible for the observed cleft palate phenotype.

## Discussion

Gene deletion studies in mice have shown that BMP signaling is required for patterning and development of most tissues and organs of the orofacial region [6], [7]. Specifically, *Bmp7*-deficient mice exhibit a variety of orofacial malformations including anophthalmia, mandible anomalies, missing or hypoplastic teeth, and cleft palate [21]. A frameshift mutation in the *BMP7* gene has been identified in humans showing a similar combination of symptoms [20]. This indicates that the *Bmp7^Δ/Δ^* mouse provides an adequate animal model for studying this apparently novel type of craniofacial syndrome. However, the etiology of these orofacial manifestations is not yet investigated, and therefore in the present study we focused on the causes of the cleft palate phenotype due to absence of Bmp7. No obvious differences in the size and shape of vertical palatal shelves at E13.5 between wildtype and *Bmp7^Δ/Δ^* embryos were observed, but at E14.5 shelf elevation above the tongue appeared delayed. One day later, shelves in *Bmp7^Δ/Δ^* embryos were still incompletely reoriented and never joined at the midline, resulting in a cleft of the secondary palate. Nevertheless, the presumptive middle edge epithelia of mutant palatal shelves were competent to fuse when placed into close apposition in tissue culture. Genes specifying distinct domains of the palatal epithelium (*Col14*, *Smoc2*) were normally expressed, as well as components known to be functionally involved in palatal fusion (*Tgfb3* [32], [35], *Mmp13* [41], *Jag2* [36]). Thus, the defect in *Bmp7^Δ/Δ^* embryos did not appear to be caused by failure of the palatal epithelium to differentiate and acquire fusion competence, but rather by a disturbed reorientation of the shelves into a horizontal position above the tongue.

Recent evidence indicates that shelf elevation is a complicated process involving extensive reorganization of the palatal mesenchyme [42], [43]; see next paragraphs). Moreover, it is well known that in its course the developing tongue has to move out of the way of the reorienting shelves. This occurs at the same developmental stage by a combination of longitudinal growth of the mandible and concomitant flattening of the tongue, [44], and the first swallowing movements of the embryo [45], [46]. Mandibular growth retardation (micrognathia) is a feature of many orofacial abnormalities in humans, such as of Stickler syndrome, which is caused by mutations in cartilage collagens II and XI [47], and of the related Pierre Robin sequence that includes cleft palate [47], [48]. Meckel's cartilage, the precursor structure in the mandible, is thought to provide an early attachment point for the tongue and to assist its elongation and flattening, thus facilitating palatal shelf elevation [49]. Micrognathia with associated cleft palate has been observed in mouse mutant strains deficient for the same cartilage collagen genes [50], [51] as well as various other genes, such as the methyltransferase *Pdrm16,* the homeobox gene *Hoxa-2*, the transcription factor *Snail*, the Activin Receptor Type I *Alk2*, epidermal growth factor receptor *Egfr* and its ligand *Tgfa* [19], [52], [53], [54], [55]. Collagen and matrix metalloproteinase (MMP) production is known to be induced by EGF signaling and crucial for development of Meckel's cartilage; defects in this entire pathway can obviously lead to underdeveloped mandible and cleft palate. Similarly, *Bmp7* that is expressed anterior to the tip of Meckel's cartilage and in the mandible, is known to regulate genes for extracellular matrix (ECM) components [56]. *Bmp7*-deficient embryos have a significantly reduced length of Meckel's cartilage, with lack of fusion at its anterior tip and failure to form the rostral process. Consequently, we observed a delayed flattening of the tongue at the time crucial for reorientation of the palatal shelves, and at the same stage mutant embryos had a protruding tongue and retruded mandible. Interestingly, mice with neural crest specific deletion of *Alk2* show unfused Meckel's cartilage and cleft palate as well [19], which underlines the connection between these two developmental processes and the role of BMP signaling in them. Thus obstruction by a malformed tongue, as a consequence of micrognathia, might be one relevant cause for palatal cleft formation in the *Bmp7^Δ/Δ^* mouse.

However, this argument does not entirely explain the etiology of cleft palate in this model. First, it is difficult to establish a formal causality between short mandible and cleft palate in many micrognathia mutants including *Bmp7^Δ/Δ^*, since the respective genes are also expressed in the developing palatal shelves themselves [19], [53], [54], [55]. Subtle differences in phenotype between micrognathia mouse strains support this notion. For example, although *Snai1^+/−^Snai2^−/−^* embryos exhibit an undersized Meckel's cartilage, shelf elevation and apposition occur normally, and in these mice cleft palate eventually results from failed fusion of the shelves [55]. In the case of *Bmp7^Δ/Δ^* embryos, we observed that in addition to micrognathia they also have shorter snouts than control littermates. More importantly, when we dissected maxillary regions of mutant embryos at E13.5 and placed them in tissue culture, we observed that, although tongue and mandible had been removed and could not interfere with shelf reorientation, palatal shelf elevation was still abnormal and delayed compared to that of explants from control embryos. This result clearly demonstrates that *Bmp7^Δ/Δ^* palatal shelves have an intrinsic defect in tissue reorganization at E13.5-E14.5, which is likely to contribute to cleft formation.

Compared to the mechanism of palatal shelf fusion, relatively little is known about the process of shelf elevation. In wild-type mouse embryos, it occurs within just a few hours around E14.5-E15. At this stage, asymmetrically elevated shelves are sometimes observed in wildtype embryos [57], and frequently in mutants with delayed reorientation, such as in *Zfxh1^−/−^* [34] or *Bmp7^Δ/Δ^* mice [21]. This indicates that shelf elevation can be slightly asynchronous, i.e. one shelf might lift shortly before the other. Originally it has been proposed that the shelves simply rotate by 90°, driven by hyaluronic acid-generated osmotic pressure [1], [58], [59]. Other studies point to a more complex mechanism of tissue reorientation and remodeling within the shelves [42], [43]. First, carbon marking studies in wildtype mouse embryos demonstrated that the vertical palatal shelves undergo a rotating movement only in the mid-palate region, whereas their anterior and posterior parts bulge out horizontally towards the tongue [43]. In the following it was shown that in the *Zfxh1^−/−^* mutant, where shelf elevation is delayed by one day, marker genes for medial edge epithelial cells such as *Mmp13* were not expressed at the distal tips of the still vertical shelves at E14.5, but rather at their distal-medial (lingual) aspects [42]. Likewise, in *Bmp7^Δ/Δ^* embryos we found *Tgfb3* expression to be confined to the epithelium of the inner distal surfaces of vertical shelves at E14.5, indicating that these epithelial domains are destined to become the midline epithelial seam. The delayed palatal shelf elevation in *Bmp7^Δ/Δ^* embryos also revealed that tissue remodeling by matrix metalloproteinases starts at the proximal-nasal corner of the shelves prior to their elevation and not as a consequence [60]. Thus, mouse mutants such as *Zfxh1^−/−^* and *Bmp7^Δ/Δ^* allow us to learn more about the complex process of palatal shelf reorganization and the genes and molecules involved.

Although our present data clearly demonstrate a role for BMP7 in morphogenesis of the mandible and the palatal shelves, the Bmp7 target genes in these processes remain to be identified. In palatal shelves of *Bmp7^Δ/Δ^* embryos, we could not detect changes in cell proliferation or apoptosis, and several mesenchymal genes known to be involved in palatogenesis exhibited a normal expression pattern. In a preliminary screen for genes involved in tissue remodeling, we found that tenascin-W was under- or misexpressed in *Bmp7^Δ/Δ^* palatal shelves at E13.5 (i.e. before elevation). Tenascin-W is a “matricellular” protein and a component of pre-osteogenic areas, and is induced by purified Bmp7 in cultured embryonic cranial fibroblasts [61]. One function of BMP7 thus might be to provide the appropriate extracellular environment for tissue reorganization. Clearly additional molecular or cellular processes are regulated by BMP7, and without their characterization it will be difficult to establish a causal link between BMP7 deficiency and the development of specific malformations such as micrognathia and cleft palate.
